# Supplementary material for: The Effect of Epimedium Isopentenyl Flavonoids on the Broiler Gut Health Using Microbiomic and Metabolomic Analyses
Source: Int J Mol Sci. 2023 Apr 21;24(8):7646. doi: 10.3390/ijms24087646 (PMC10141048; doi:10.3390/ijms24087646)
Supplement: Supplementary file 1 [file ijms-24-07646-s001.zip › ijms-2277135-supplementary.pdf]

The effect of Epimedium isopentenyl flavonoids on the broiler gut health using microbiomic and metabolomic analyses

Jiaqi Zhang[1,2], Qingyu Zhao[1,2], Wei Si[1,2], Yuchang Qin[1,2], Huiyan Zhang[1,2*] and Junmin Zhang[1,2*]
[1]State Key Laboratory of Animal Nutrition, Institute of Animal Sciences, Chinese Academy of Agricultural Sciences, Beijing 100193, China
[2] Scientific Observing and Experiment Station of Animal Genetic Resources and Nutrition in North China of Ministry of Agriculture and Rural Affairs, Institute of Animal Sciences of Chinese Academy of Agricultural Sciences, Beijing 100193, China

Supplementary Material

Supp. Information1: Qualitative analysis of the main isopentenyl flavonols components of EM by UHPLC-Q-TOF/MS.
Supp. Information 2: Quantification of the main isopentenyl flavonol components of EM by UHPLC-QqQ-MS/MS.
Supp. Information 3: Quantification of cecal lactic acid by UHPLC-QqQ-MS/MS.
Supp. Information 4: Details of the 16S rRNA high-throughput sequencing.
Supp. Information 5: Details of the UHPLC-Q Exactive HF-X system for the detection of serum metabolites.
Figure S1. The total ion current profiles on positive ions mode (A) and negative ions mode (B) and DAD chromatography (C) of Epimedium extracts for UHPLC-Q-TOF/MS detection.
Figure S2. The top five vip value metabolites Pantothenic Acid (A), L-Methionine (B), 1-(1,2,3,4,5-Pentahydroxypent-1-yl)-1,2,3,4-tetrahydro-beta-carboline-3-carboxyl (C), 5-(3',5'-Dihydroxyphenyl)-gamma-valerolactone-O-sulphate-O-methyl (D), Pyrocatechol sulfate (E). Significant differences were recorded by * $0.01 < p \leq 0.05$, ** $0.001 < p \leq 0.01$, *** $p \leq 0.001$. N = 6 per treatment group.
Figure S3. (A) KEGG topology analysis of differential serum metabolites between the CTC and NC groups. (B) KEGG topology analysis of differential serum metabolites between the EM and NC groups.
Figure S4. Indicates correlation between serum metabolites and cecum microbes of the first 30 (genus level) that changed significantly between the treatment groups.
Table S1. Composition and nutrient levels of experimental diet (%, air-dry basis).
Table S2. Total of 48 serum metabolites changed significantly (up and down) after screening that the fold-change value was > 2, and the p-value was < 0.05.

Supp. Information1: Qualitative analysis of the main isopentenyl flavonol components of EM by UHPLC-Q-TOF/MS.

UHPLC-Q-TOF/MS was used for qualitative analysis of the main components of EM. The chromatographic separation was performed on Aglient InfinityLab Poroshell 120 SB-C18 column, 2.7 μm particle size and 2.1 × 100 mm i.d. column (Sigma Aldrich, Saint Louis, MO, USA). Aqueous formic acid (0.1% v/v) solution (A) and acetonitrile solution with 0.1% formic acid (B) consisted of mobile phase, with a flow rate of 0.3 mL/min, under gradient elution at 40 °C. The gradient profile was as follows: 1–3 min, 5–20% B; 3– 17 min, 20–100% B; 17–22 min, 100% B; 22–22.1 min, 100–5% B; 22.1–23 min, 5% B.

TOF-MS: electrospray ionization source (ESI), scanning mode: positive and negative ions. The MS parameters are as follows: dry gas temperature: 325°C; dry gas flow rate: 6 L/min; desolvent gas flow rate: 800 L/h; capillary voltage: 3.0 kV; collision-induced dissociation voltage: 500 V; collision energy: 20–50 eV; atomizer pressure: 350 psi; auxiliary gas: N_2; positive and negative reference ion calibration ($[M+H]^+$ = 121.0509 and 922.0098, $[M-H]^-$ = 112.9859 and 1033.9881) to ensure accuracy in spectral acquisition. The range of data acquisition is 100 to 1000.

Supp. Information 2: Quantification of the main isopentenyl flavonol components of EM by UHPLC-QqQ-MS/MS.

UHPLC-QqQ-MS/MS quantitative analysis

The flavonoid profile analysis was performed using a UHPLC system model 1290 Infinity coupled to a 6545 Triple Quadrupole mass spectrometer (UHPLC-QqQ-MS/MS) equipped with an electrospray ionization (Agilent Technologies, Palo Alto, USA). The chromatographic separation was performed on Aglient InfinityLab Poroshell 120 SB-C18 column, 2.7 μm particle size and 2.1 × 100 mm column (Sigma Aldrich, Saint Louis, MO, USA). Aqueous formic acid (0.1% v/v) solution (A) and acetonitrile solution with 0.1% formic acid (B) consisted of mobile phase, with a flow rate of 0.3 mL/min, under gradient elution at 40 °C. The gradient profile was as follows: 0–6 min, 25% B; 6–10 min, 30 % B; 10–12 min, 30-52% B; 12–17 min, 52% B. The injection volume was 10 μL. The LC-MS data were acquired using Mass Hunter software (Version 10.0, Agilent Technologies).

Mass spectrometry conditions were as follows: source temperature: 350 °C; gas flow: 6 L/h; nebulizer: 45 psi; sheath gas temperature: 300 °C; sheath gas flow: 10 L/min; capillary: 3500 V; nozzle: 500 V; acceleration cell voltage: 5 V; dwell time: 9.8 ms. Two selected reaction monitoring (SRM) transitions were optimized for each compound identified. The optimal multiple reaction monitoring (MRM) parameters for the target compounds were listed in Table 2.

Supp. Information 3: Quantification of cecal lactic acid by UHPLC-QqQ-MS/MS.
The cecal lactic acid analysis was performed using a UHPLC system model 1290 Infinity coupled to a 6545 Triple Quadrupole mass spectrometer (UHPLC-QqQ-MS/MS) equipped with an electrospray ionization (Agilent Technologies, Palo Alto, USA). The chromatographic separation was performed on T3 C18 column (Waters, USA). Aqueous formic acid (0.1% v/v) solution (A) and methanol (B) consisted of mobile phase, with a flow rate of 0.3 mL/min, under gradient elution at 40 °C. The gradient profile was as follows: 0–1 min, 3% B; 1–2.5 min, 3-70 % B; 2.5–4 min, 70-3% B; 4–5 min, 3% B. The injection volume was 1 μL. The LC-MS data were acquired using Mass Hunter software (Version 10.0, Agilent Technologies).
Mass spectrometry conditions were as follows: source temperature: 350 °C; gas flow: 6 L/h; nebulizer: 45 psi; sheath gas temperature: 300 °C; sheath gas flow: 10 L/min; capillary: 3500 V; nozzle: 500 V; acceleration cell voltage: 5 V; dwell time: 9.8 ms. Lactic acid was quantified by MS in negative ion mode, a quantifier ion transition m/z 89.0 → 45.0 and a qualifier 89.0 → 43.1 were used with a fragmentation voltage of 50 V, collision energy of 8 V, and cell accelerator voltage of 5 V.

Supp. Information 4: Details of the 16S rRNA high-throughput sequencing.
Total genomic DNA from the cecum samples was extracted by QIAamp DNA Stool Mini Kit (Qiagen, Hilden, Germany). DNA concentration and integrality were detected by NanoDrop Spectrophotometer (Thermo Scientific, Wilmington, DE, USA) and agarose gel electrophoresis, respectively. DNA concentration of each cecal content was diluted to 10 ng/μL using double-distilled water. The V3–V4 region of 16S rDNA was amplified using the following specific primers (338F: 5′-ACTCCTACGGGAGGCAGCAG-3′; 806R: 5′-GGACTACHVGGGTWTCTAAT-3′). Purified amplicons were pooled in equal amounts and paired-end sequenced (2×250 bp) on an Illumina MiSeq platform at Majorbio Bio-Pharm Technology Co. Ltd. (Shanghai, China).
The raw data was uploaded to the NCBI Sequence Read Archive databas (Accession Number: PRJNA904521). QIIME2 (version 2021.8 or higher, http://qiime2.org) was used for quality control and analysis of sequence reads. Raw fastq files were demultiplexed using q2-demux and quality filtered and dereplicated with q2-dada2. Sequences with average Phred scores below 25 were removed. The amplified subsequence variants (ASVs) were classified using the q2-feature classifier classify-sklearn naïve Bayes classifier on the SILVA database version 132 (https://www.arb-silva.de/download/archive/qiime/). MAFFT was used for sequence comparison of multiple ASVs by q2-alignment, and phylogeny was constructed by q2-phylogeny using fasttree2. To calculate α- and β-diversity, data were diluted to the lowest number of sequences possible for each sample. α-diversity was measured by the Shannon index. differences between α-diversity indices were tested by the Kruskal-Wallis test (QIIME 2). To estimate the similarity of microbial community structure (β-diversity) between groups, a principal coordinate analysis (PCoA) based on a weighted UniFrac distance matrix (QIIME 2) was performed. Partial least squares discriminant analysis (PLS-DA) was conducted to compare the bacterial community structures across all samples. Moreover, the significance of differentiation of microbial structure among groups was statistically tested by analysis of similarity. The linear discriminant analysis (LDA) was coupled with effect size measurements (LEf-Se) to distinguish the bacteria between all the treatments, the LDA score was set at two. To conduct functional prediction analysis of the 16S data, data analysis was performed through the Phylogenetic Investigation of Communities by Reconstruction of Unobserved States (PICRUSt)2 pipeline.

Supp. Information 5: Details of the UHPLC-Q Exactive HF-X system for the detection of serum metabolites.

Chromatographic conditions:

2μL of sample was separated by HSS T3 column (100 mm × 2.1 mm i.d., 1.8 μm) and then entered into mass spectrometry detection. The mobile phases consisted of 0.1% formic acid in water:acetonitrile (95:5, v/v) (solvent A) and 0.1% formic acid in acetonitrile:isopropanol:water (47.5:47.5:5, v/v)(solvent B). The solvent gradient changed according to the following conditions: from 0 to 3.5 min, 0% B to 24.5% B (0.4 mL/min); from 3.5 to 5 min, 24.5% B to 65% B (0.4 mL/min); from 5 to 5.5 min, 65% B to 100% B (0.4 mL/min); from 5.5to 7.4 min, 100% B to 100% B (0.4 mL/min to 0.6 mL/min); from 7.4 to 7.6 min, 100% B to 51.5% B (0.6 mL/min); from 7.6 to 7.8 min, 51.5% B to 0% B (0.6 mL/min to 0.5 mL/min); from 7.8 to 9 min, 0% B to 0% B (0.5 mL/min to 0.4 mL/min);from 9 to 10 min, 0% B to 0% B (0.4 mL/min) for equilibrating the systems. The sample injection volume was 2 μL and the flow rate was set to 0.4 mL/min. The column temperature was maintained at 40 ℃. During the period of analysis, all these samples were stored at 4 ℃.

MS conditions:

The mass spectrometric data was collected using a Thermo UHPLC -Q Exactive HF-X Mass Spectrometer equipped with an electrospray ionization (ESI) source operating in either positive or negative ion mode. The optimal conditions were set as followed: heater temperature, 425 ℃ ; Capillary temperature, 325 ℃; sheath gas flow rate, 50 arb; Aux gas flow rate, 13 arb; ion-spray voltage floating (ISVF),-3500V in negative mode and 3500V in positive mode, respectively; Normalized collision energy , 20-40-60V rolling for MS/MS. Full MS resolution was 60000, and MS/MS resolution was 7500. Data acquisition was performed with the Data Dependent Acquisition (DDA) mode. The detection was carried out over a mass range of 70-1050 m/z.

Figure S1. The total ion current profiles on positive ions mode (A) and negative ions mode (B) and DAD chromatography (C) of Epimedium extracts for UHPLC-Q-TOF/MS detection.

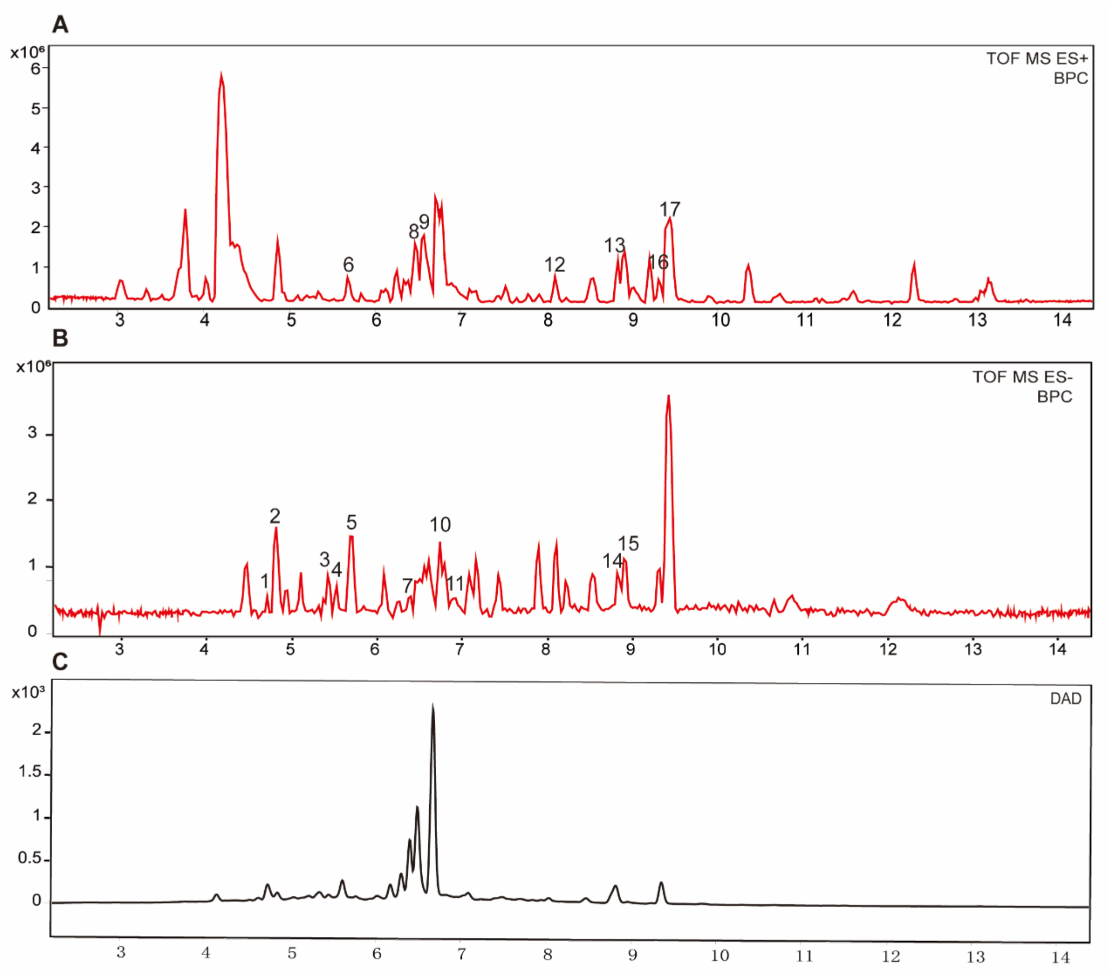

Figure S2. The top five vip value metabolites Pantothenic Acid (A), L-Methionine (B), 1-(1,2,3,4,5-Pentahydroxypent-1-yl)-1,2,3,4-tetrahydro-beta-carboline-3-carboxyl (C), 5-(3’,5’-Dihydroxyphenyl)-gamma-valerolactone-O-sulphate-O-methyl (D), Pyrocatechol sulfate (E). Significant differences were recorded by $0.01 < P \leqslant 0.05$*, $0.001 < P \leqslant 0.01$**, $P \leqslant 0.001$***. N = 6 per treatment group.

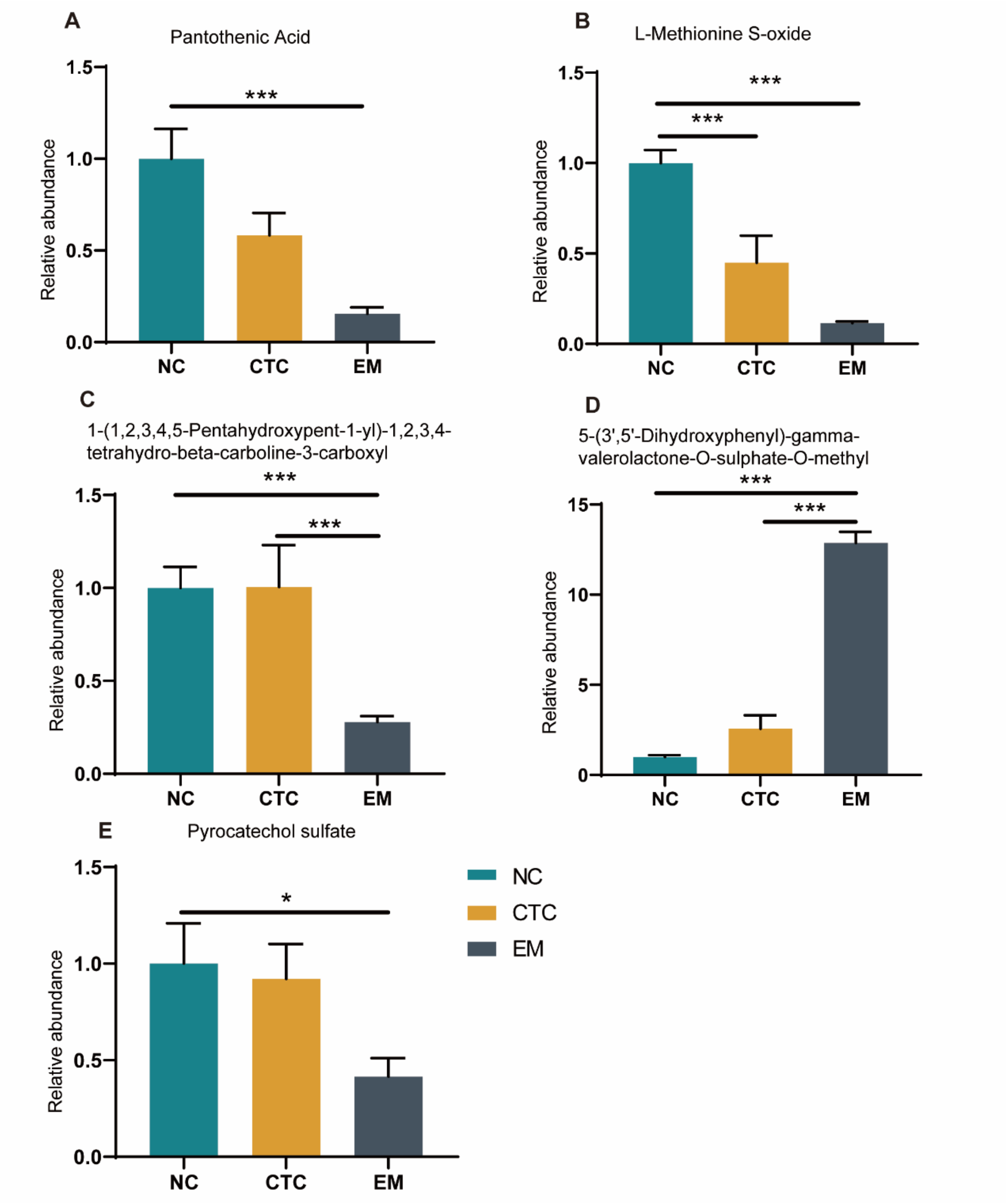

Figure S3. (A) KEGG topology analysis of differential serum metabolites between the CTC and NC groups. (B) KEGG topology analysis of differential serum metabolites between the EM and NC groups.

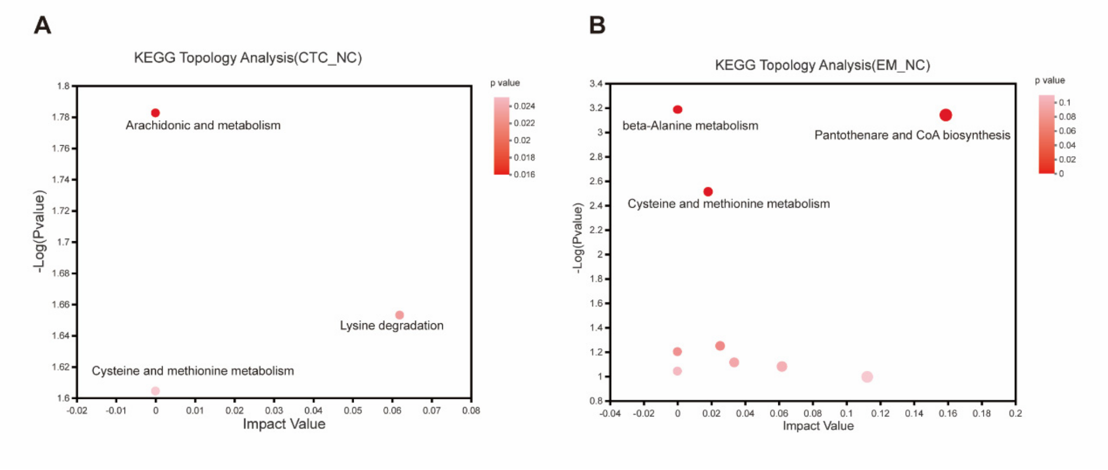

Figure S4. Indicates correlation between serum metabolites and cecum microbes of the first 30 (genus level) that changed significantly between the treatment groups.

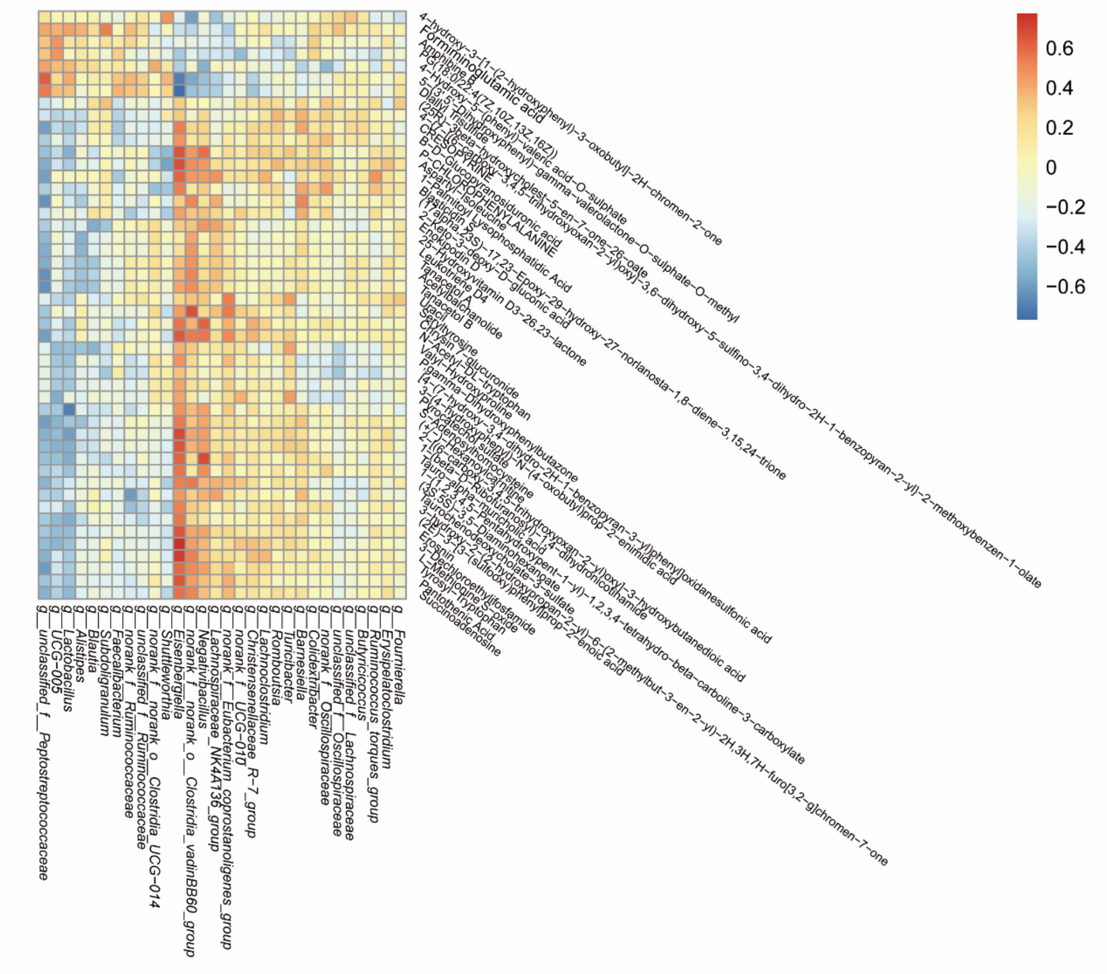

Supp. Table S1. Composition and nutrient levels of experimental diet (%, air-dry basis).

Items	Starter phase (d 1 to 21)	Grower phase (d 22 to 42)
Ingredients		
Corn	55.03	58.58
Soybean meal	32.5	31.7
Fish meal	4.3	1.8
Soybean oil	4.2	4
Dicalcium phosphate	1.45	1.7
Limestone	1.34	1.15
DL-methionine	0.23	0.12
Sodium chloride	0.3	0.3
Choline chloride	0.15	0.15
Premix[1]	0.5	0.5
Nutrient levels		
Metabolizable energy, MJ/kg	12.77	12.98
Crude protein, %	21.59	20.02
Calcium, %	1.07	0.94
Total phosphorus, %	0.72	0.69
Available phosphorus, %	0.45	0.41
Lysine, %	1.21	1.08
Methionine+Cysteine, %	0.94	0.78
Threonine, %	0.83	0.77
Tryptophan, %	0.25	0.24

[1]*Premix supplied per kg of diet: vitamin A, 10,000 IU, vitamin D_3, 3,200 IU, vitamin E, 15 IU, vitamin K_3, 5 mg, thiamin, 2.1 mg, riboflavin, 7 mg, vitamin B_{12}, 0.02 mg, biotin, 0.17 mg, folic acid, 1.5 mg, iron (from $FeSO_4{\cdot}7H_2O$), 80 mg; copper (from $Cu_2(OH)_3Cl$), 20 mg; zinc (from $ZnSO_4$ H_2O), 90 mg; manganese (from $MnSO_4$ $\cdot H_2O$), 80 mg; iodine (from KI), 0.4 mg; selenium (from Na_2SeO_3), 0.4 mg.*

Supp. Table S2. Total of 48 serum metabolites changed significantly (up and down) after screening that the fold-change value was>2, and the *P*-value was<0.05 [1].

No	Metabolite	Formula	Related category	RT (min)	Mass (m/z)	Ion mode	CTC VS NC		EM VS NC	
							Trend	Fold change	Trend	Fold change
1	Pantothenic Acid	$C_9H_{17}NO_5$	Organic oxygen compounds	2.93	220.12	$[M+H]^+$	down[2]	0.583	down	0.155
2	Valyl-Hydroxyproline	$C_{10}H_{18}N_2O_4$	Organic acids and derivatives	2.85	231.13	$[M+H]^+$	down	0.593	down	0.397
3	L-Methionine S-oxide	$C_5H_{11}NO_3S$	Organic acids and derivatives	1.22	148.04	$[M+H]^+$	down	0.448	down	0.115
4	Tauro-alpha-muricholic acid	$C_{26}H_{45}NO_7S$	Lipids and lipid-like molecules	6.22	533.33	$[M+H]^+$	down	0.835	down	0.191
5	P,gamma-Dihydroxyphenylbutazone	$C_{19}H_{20}N_2O_4$	other	6.1	341.15	$[M+H]^+$	down	0.506	down	0.096
6	CRESOPYRINE	$C_{10}H_{10}O_4$	other	2.78	212.09	$[M+H]^+$	down	1.036	down	0.476
7	1-(1,2,3,4,5-Pentahydroxypent-1-yl)-1,2,3,4-tetrahydro-beta-carboline-3-carboxylate	$C_{17}H_{22}N_2O_7$	other	3.16	367.15	$[M+H]^+$	down	1.005	down	0.279
8	3-(4-hydroxyphenyl)-N-(4-oxobutyl)prop-2-enimidic acid	$C_{13}H_{15}NO_3$	other	3.49	251.14	$[M+H]^+$	down	0.623	down	0.388
9	5-(3',5'-Dihydroxyphenyl)-gamma-valerolactone-O-sulphate-O-methyl	$C_{12}H_{14}O_7S$	Organic acids and derivatives	1.12	347.02	$[M+H]^+$	up[3]	2.561	up	12.862
10	Erosnin	$C_{18}H_8O_6$	Phenylpropanoids and polyketides	1.62	359	$[M+H]^+$	down	0.519	down	0.162

11	3-Dechloroethylifosfamide	$C_5H_{12}ClN_2O_2P$	other	1.72	397.07	$[M+H]^+$	down	0.542	down	0.100
12	2-[(6-carboxy-3,4,5-trihydroxyoxan-2-yl)oxy]-3-hydroxybutanedioic acid	$C_{10}H_{14}O_{12}$	other	2.35	371.02	$[M+H]^+$	down	0.614	down	0.263
13	Uracil	$C_4H_4N_2O_2$	Organoheterocyclic compounds	2.35	113.04	$[M+H]^+$	down	0.606	down	0.499
14	Blasticidin S	$C_{17}H_{26}N_8O_5$	Organic acids and derivatives	2.76	486.22	$[M+H]^+$	down	0.740	down	0.340
15	B-D-Glucopyranosiduronic acid	$C_{15}H_{21}NO_8$	other	2.77	344.13	$[M+H]^+$	up	1.413	down	0.332
16	Succinoadenosine	$C_{14}H_{17}N_5O_8$	Nucleosides, nucleotides, and analogues	2.93	384.12	$[M+H]^+$	down	0.634	down	0.234
17	Tyrosyl-Tryptophan	$C_{20}H_{21}N_3O_4$	Organic acids and derivatives	3.26	409.19	$[M+H]^+$	down	0.515	down	0.099
18	(+/-)-Hexanoylcarnitine	$C_{13}H_{25}NO_4$	other	4.44	260.19	$[M+H]^+$	down	0.333	down	0.118
19	Chrysin 7-glucuronide	$C_{21}H_{18}O_{10}$	other	4.91	431.1	$[M+H]^+$	down	0.879	down	0.225
20	P-CHLOROPHENYLALANINE	$C_9H_{10}ClNO_2$	Phenylpropanoids and polyketides	5.85	200.05	$[M+H]^+$	down	0.595	down	0.476
21	Acetylbalchanolide	$C_{17}H_{24}O_4$	Lipids and lipid-like molecules	6.06	310.2	$[M+H]^+$	down	0.350	down	0.198
22	(3S,5S)-3,5-Diaminohexanoate	$C_6H_{14}N_2O_2$	Organic acids and derivatives	6.6	111.09	$[M+H]^+$	down	0.485	down	0.313
23	Amphibine B	$C_{39}H_{47}N_5O_5$	other	6.97	666.37	$[M+H]^+$	down	0.750	up	2.353
24	PG(18:0/22:4(7Z,10Z,13Z,16Z))	$C_{46}H_{83}O_{10}P$	Lipids and lipid-like molecules	7.87	865.53	$[M+H]^+$	up	1.267	up	2.009

25	Diallyl Trisulfide	$C_6H_{10}S_3$	Organosulfur compounds	9.99	179	$[M+H]^+$	up	1.262	up	5.023
26	(17alpha,23S)-17,23-Epoxy-29-hydroxy-27-norlanosta-1,8-diene-3,15,24-trione	$C_{29}H_{40}O_5$	Lipids and lipid-like molecules	6.68	507.25	$[M+H]^+$	down	0.701	down	0.497
27	25-Hydroxyvitamin D3-26,23-lactone	$C_{27}H_{40}O_4$	Lipids and lipid-like molecules	6.67	492.31	$[M+H]^+$	down	0.632	down	0.491
28	Leukotriene D4	$C_{25}H_{40}N_2O_6S$	Lipids and lipid-like molecules	6.67	514.29	$[M+H]^+$	down	0.448	down	0.293
29	Tanacetol B	$C_{17}H_{28}O_4$	Lipids and lipid-like molecules	6.22	314.23	$[M+H]^+$	down	0.402	down	0.210
30	Tanacetol A	$C_{17}H_{26}O_4$	Lipids and lipid-like molecules	6.12	312.22	$[M+H]^+$	down	0.396	down	0.206
31	N-Acetyl-DL-tryptophan	$C_{13}H_{14}N_2O_3$	other	5.09	247.11	$[M+H]^+$	down	0.473	down	0.100
32	Seryltyrosine	$C_{12}H_{16}N_2O_5$	Organic acids and derivatives	4.25	251.1	$[M+H]^+$	down	0.352	down	0.146
33	Enokipodin D	$C_{15}H_{18}O_4$	Lipids and lipid-like molecules	3.6	280.16	$[M+H]^+$	down	0.302	down	0.328
34	1-(beta-D-Ribofuranosyl)-1,4-dihydronicotinamide	$C_{11}H_{16}N_2O_5$	Organic oxygen compounds	2.75	257.11	$[M+H]^+$	down	0.709	down	0.316
35	3-hydroxy-2-(2-hydroxypropan-2-yl)-6-(2-methylbut-3-en-2-yl)-2H,3H,7H-furo[3,2-g]chromen-7-one	$C_{19}H_{22}O_5$	other	2.74	375.12	$[M+H]^+$	down	1.028	down	0.375

36	(2E)-3-[3-(sulfooxy)phenyl]prop-2-enoic acid	$C_9H_8O_6S$	Phenylpropanoids and polyketides	2.53	262.04	+	down	0.893	down	0.313
37	S-Adenosylhomocysteine	$C_{14}H_{20}N_6O_5S$	Organoheterocyclic compounds	2.46	385.13	$[M+H]^+$	down	0.948	down	0.357
38	Aspartyl-Isoleucine	$C_{10}H_{18}N_2O_5$	Organic acids and derivatives	2.28	229.12	$[M+H]^+$	down	0.678	down	0.315
39	4-{7-[(6-carboxy-3,4,5-trihydroxyoxan-2-yl)oxy]-3,6-dihydroxy-5-sulfino-3,4-dihydro-2H-1-benzopyran-2-yl}-2-methoxybenzen-1-olate	$C_{22}H_{24}O_{14}S$	other	2.28	527.08	$[M+H]^+$	up	1.708	down	0.221
40	4-hydroxy-3-[1-(2-hydroxyphenyl)-3-oxobutyl]-2H-chromen-2-one	$C_{19}H_{16}O_5$	other	1.01	347.09	$[M+H]^+$	up	3.495	up	2.175
41	Taurochenodeoxycholate-3-sulfate	$C_{26}H_{45}NO_9S_2$	Lipids and lipid-like molecules	6.4	288.62	$[M-H]^-$	down	0.707	down	0.129
42	Pyrocatechol sulfate	$C_6H_6O_5S$	Organic acids and derivatives	3.23	188.99	$[M-H]^-$	down	0.922	down	0.415
43	[4-(7-hydroxy-3,4-dihydro-2H-1-benzopyran-3-yl)phenyl]oxidanesulfonic acid	$C_{15}H_{14}O_6S$	other	5.73	321.04	$[M-H]^-$	down	0.902	down	0.485
44	1-Palmitoyl Lysophosphatidic Acid	$C_{19}H_{39}O_7P$	other	7.12	409.23	$[M-H]^-$	down	0.674	down	0.487
45	(25R)-3beta-hydroxycholest-5-en-7-one-26-oate	$C_{27}H_{42}O_4$	Lipids and lipid-like molecules	6.65	429.3	$[M-H]^-$	up	1.258	down	0.385

46	4-Hydroxy-5-(phenyl)-valeric acid-O-sulphate	$C_{11}H_{14}O_6S$	Benzenoids	6.06	255.03	$[M-H]^-$	down	6.891	up	89.008
47	Formiminoglutamic acid	$C_6H_{10}N_2O_4$	Organic acids and derivatives	1.1	173.06	$[M-H]^-$	up	1.315	up	2.214
48	2-Keto-3-deoxy-D-gluconic acid	$C_6H_{10}O_6$	Organic acids and derivatives	1.09	177.04	$[M-H]^-$	down	0.729	down	0.416

[1]NC: basal diet; CTC: basal diet + 75 mg/kg chlortetracycline; EM: basal diet + 200 mg/kg epimedium extract. [2]The distinguished metabolite was down-regulated in the CTC group compared to the NC group. [3]The distinguished metabolite was up-regulated in the EM group compared to the NC group.
